# Supplementary material for: Splice-Junction-Based Mapping of Alternative Isoforms in the Human Proteome
Source: Cell Rep. Author manuscript; Available in PMC 2020 Jan 15. (PMC6961840; doi:10.1016/j.celrep.2019.11.026)
Supplement: 3 [file NIHMS1546469-supplement-3.zip › DF2/PXD006675/SmoothMuscleCells-64-Q96B23-TITAEIPGHLDPGFLASDK.pdf]

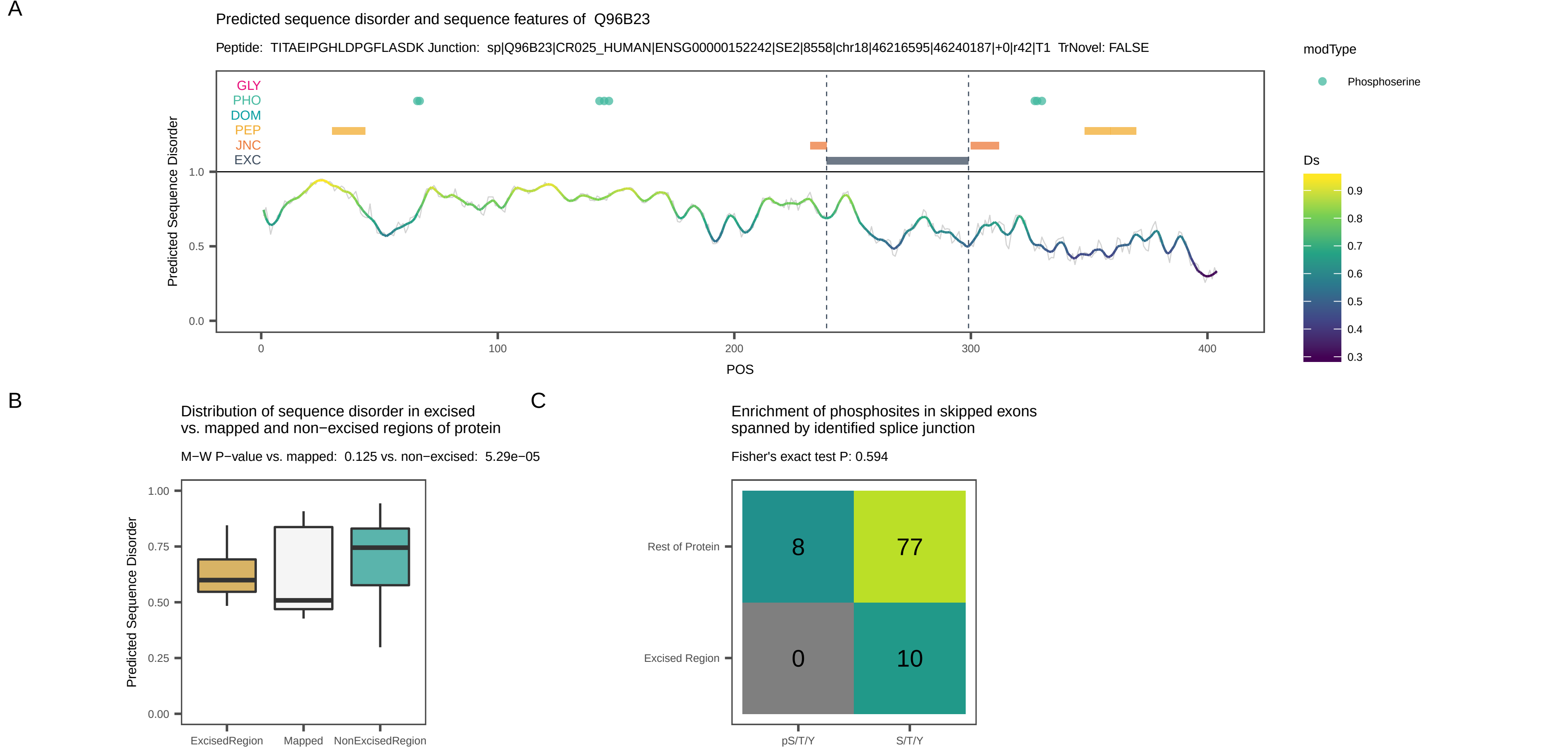
A
Predicted sequence disorder and sequence features of Q96B23
Peptide: TITAEIPGHLDPGFLASDK Junction: sp|Q96B23|CR025_HUMAN|ENSG00000152242|SE2|8558|chr18|46216595|46240187|+0|r42|T1 TrNovel: FALSE
GLY
PHO
DOM
PEP
JNC
EXC
Predicted Sequence Disorder
1.0
0.5
0.0
0
100
200
300
400
POS
modType
Phosphoserine
Ds
0.9
0.8
0.7
0.6
0.5
0.4
0.3
B
Distribution of sequence disorder in excised
vs. mapped and non−excised regions of protein
M−W P−value vs. mapped: 0.125 vs. non−excised: 5.29e−05
Predicted Sequence Disorder
1.00
0.75
0.50
0.25
0.00
ExcisedRegion
Mapped
NonExcisedRegion
C
Enrichment of phosphosites in skipped exons
spanned by identified splice junction
Fisher's exact test P: 0.594
Rest of Protein
Excised Region
8
77
0
10
pS/T/Y
S/T/Y
